# Immediate effects of spinal manipulation, exercise therapy, and open-label placebo on lifting performance and pain in individuals with chronic non-specific back pain: a motion laboratory-based crossover randomized controlled trial

**DOI:** 10.1186/s12998-026-00680-w

**Published:** 2026-10-14

**Authors:** Fredrik Borg, Filip Gedin, Erika Franzén, Wilhelmus Johannes Andreas Grooten

**Affiliations:** 1 Department of Neurobiology, Care Sciences and Society, Division of Physiotherapy, Karolinska Institutet, Alfred Nobels Allé 23, Huddinge, Stockholm, 141 83 Sweden; 2 Scandinavian College of Chiropractic, Campus Solna, Fogdevreten 2A, 171 65 Solna, Stockholm, Sweden; 3 Department of Clinical Neuroscience, Karolinska Institutet, Stockholm, Sweden; 4 Medical Unit Allied Health Professionals, Karolinska University Hospital, Women’s Health and Allied Health Professionals Theme, Stockholm, Sweden; 5 School of Health and Welfare, Dalarna University, Falun, SE-791 88 Sweden

**Keywords:** Spinal manipulative therapy, Motion capture, Movement quality, Biomechanics, Physical function, Everyday activities, Functional data analysis, Intersegmental coordination, Placebo effects

## Abstract

**Background:**

Chronic non-specific back pain (CBP) is a leading cause of disability worldwide and is commonly associated with limitations in movements such as lifting. Although spinal manipulation (SMT) and exercise therapy (ET) are recommended treatments for CBP, their effects on objectively measured movement performance remain insufficiently explored. Open-label placebo (OLP) has gained attention for engaging placebo-related mechanisms ethically. This study aimed to investigate the immediate effects of SMT, ET, and OLP on lifting performance and pain in individuals with CBP, and to evaluate the add-on effects of OLP.

**Methods:**

A motion laboratory–based crossover randomized controlled trial included 50 individuals with CBP. Participants were recruited between April and July 2025 and attended a single laboratory session between September and October 2025. Participants performed lifting tasks using light and heavy loads before and after SMT, ET, and OLP interventions in randomized order. Three-dimensional motion capture was used to assess temporal, spatial, and spatiotemporal aspects of lifting performance. Subjective outcomes included pain intensity, perceived effectiveness, willingness to undergo each treatment, and treatment preference. Data were analyzed using linear mixed-effects models and functional data analysis.

**Results:**

Overall lifting performance changed to a small degree following the interventions. No significant between-group differences were observed at the primary endpoint. Significant within-group changes were observed following SMT, including altered intersegmental coordination during the light lift, characterized by a greater relative contribution of back movement early in the lifting phase, reduced thorax–pelvis tilt during the heavy lift, and reduced pain. OLP was associated with reduced thorax–pelvis tilt and increased hip and knee flexion during the light lift. Subjective evaluations did not consistently correspond to objective biomechanical changes.

**Conclusion:**

SMT, ET, and OLP demonstrated limited immediate effects on overall lifting performance in individuals with CBP. However, some within-group changes in thorax–pelvis movement and intersegmental coordination suggest that certain aspects of movement quality may respond to short-term interventions even when conventional biomechanical outcomes remain largely unchanged. The findings suggest the value of integrating subjective and objective outcomes, while advanced biomechanical analyses may provide insight into subtle movement changes in CBP.

**Trial registration:**

The trial is registered at ClinicalTrials.gov (ID NCT06891625).

**Supplementary Information:**

The online version contains supplementary material available at https://doi.org/10.1186/s12998-026-00680-w.

## Background

Chronic non-specific back pain (CBP) is a leading cause of disability worldwide [1] and frequently limits everyday movements, including lifting. Manual handling is a significant contributor to the development of musculoskeletal disorders [2], and the intensity and frequency of lifting have been shown to predict back pain [3]. Individuals with CBP are more likely to perceive lifting as painful and adopt compensatory movement strategies [4, 5]. Among individuals with CBP, lifting is often characterized by greater knee flexion and a more upright trunk, rather than a trunk-dominant lifting strategy [6, 7]. Although maintaining a more upright trunk during lifting is widely recommended to prevent back pain, supporting evidence for this advice is limited [6]. In contrast, asymptomatic individuals tend to use greater trunk flexion during lifting [6, 7], highlighting a paradox that challenges the traditional view of a more upright trunk as a ‘protective’ posture and raising the question of whether reduced trunk flexion represents a cause or a consequence of back pain. Impaired lifting performance can negatively affect work ability and everyday functioning. Together, these findings highlight the importance of investigating lifting performance in individuals with CBP.

Spinal manipulation (SMT) and exercise therapy (ET) are well-established interventions recommended by several clinical practice guidelines [8–11]. These interventions have been shown to improve physical function and reduce pain over the course of a treatment period [12–14]. SMT is defined as a high-velocity, low-amplitude (HVLA) thrust applied manually to the spine or related joints and is proposed to exert its effects through neurophysiological mechanisms involving peripheral, spinal, and supraspinal pathways, as well as biomechanical mechanisms [15–19]. ET commonly involves graded and individually tailored exercises and is often delivered within a biopsychosocial framework, where physical, psychological, and behavioural factors can influence treatment responses [20, 21].

Despite being widely recommended and frequently used in clinical practice, evidence regarding the immediate effects of SMT and ET on objectively measured movement performance remains limited, particularly during functional tasks such as lifting, gait, and forward bending. Most previous studies have relied on patient-reported outcome measures (PROMs) of pain and disability, whereas objective biomechanical assessments, including high-precision motion capture, remain uncommon. Furthermore, movement performance is often evaluated using discrete biomechanical outcomes such as task completion time or range of motion (ROM), providing important but partial insights into movement. In this context, movement quality represents a distinct aspect of movement performance by focusing on how movements are performed, including aspects such as movement smoothness and coordination between body segments during movement [22].

Disentangling intervention-specific effects from contextual factors, including placebo responses, remains challenging in interventions such as SMT and ET, which are difficult to blind. Contextual and non-specific factors may meaningfully contribute to treatment outcomes in CBP, further complicating the interpretation of intervention-specific effects [23, 24]. Emerging evidence suggests that open-label placebos (OLP), in which patients are fully informed they are receiving a placebo, may improve self-reported outcomes of pain and physical function in individuals with CBP [25]. OLP represents an ethical approach that engages placebo-related mechanisms without deception. Proposed mechanisms include conditioning and expectancy-related processes [26], through which expectations, prior experiences, and contextual factors may influence symptom perception and other treatment-related outcomes.

Placebo-related mechanisms may provide valuable insight into the therapeutic process, particularly in chronic pain conditions. In non-specific back pain, where symptoms are not directly attributable to clear tissue damage or identifiable disease, treatment often aims to improve function and reduce pain rather than resolve an underlying pathology. This perspective highlights the importance of contextual and interpersonal factors in treatment and supports the investigation of OLP alongside established interventions. Despite increasing interest in OLP, its effects on objectively measured physical function remain largely unexplored.

A critical gap remains in understanding how these interventions influence movement performance during everyday functional tasks. Therefore, this study aimed to investigate the immediate effects of SMT, ET, and OLP on lifting performance and pain in individuals with chronic non-specific back pain, with OLP included as an intervention in its own right, and to evaluate its potential add-on effects when combined with SMT or ET.

## Materials and methods

This study was approved by the Swedish Ethical Review Authority in Stockholm, Sweden (Dnr 2024-07604-01), conducted in accordance with the Declaration of Helsinki [27], and reported in accordance with the CONSORT 2025 statement and checklist [28]. The trial is registered at ClinicalTrials.gov (ID NCT06891625) and a study protocol was submitted in October 2025 (currently under review). All participants received written and verbal information about the study and provided written informed consent prior to inclusion.

### Study design

A motion laboratory-based crossover randomized controlled trial was conducted, in which all participants underwent all interventions and completed all outcome assessments in a randomized order during a single laboratory session lasting approximately 60 min. All interventions were delivered by the same treating chiropractor in the same motion laboratory and within the same laboratory session. Outcomes were assessed before (T0), between interventions (T1, T2), and after all interventions (T3), with post-intervention assessments conducted immediately following each intervention. This design allowed for assessment of between-group differences at T1 and within-group changes from T0 to T1 for each intervention. In addition, add-on effects of OLP were evaluated by comparing change scores following SMT and ET alone (T0–T1) with SMT and ET combined with OLP (T0–T2). Furthermore, at T3, perceived effectiveness, willingness to undergo the interventions, and treatment preference were evaluated. Outcomes included movement characteristics of the lifting tasks, gait, and forward flexion. The present article focuses on lifting characteristics, while results related to gait and forward flexion will be reported in subsequent publications.

### Participants

Participants were individuals with CBP, defined as either continuous or recurrent pain persisting for more than 3 months, aged ≥ 18 years, and able to communicate in Scandinavian languages or English. Back pain was defined as pain originating from the spine (Th1–S1), including the costovertebral and sacroiliac joints, covering both the thoracic and lumbar regions, from Th1 to the inferior gluteal fold, excluding the scapular region. Exclusion criteria included red flags [29], specific back disorders, rheumatic or inflammatory joint or spinal diseases, fractures, diagnosed hip osteoarthritis, fibromyalgia, or neurological comorbidities.

### Sample size calculation

A sample size calculation was performed using data from a previous movement laboratory study on unstable lifting in seven healthy individuals [2]. An online power calculator [30] indicated that eight participants per group would be required to detect a significant difference of 21 ms in downward phase time during the lifting task (120 vs. 99 ms, SD = 15) with α = 0.05 and 80% power. For total back motion during the upward phase, 11 participants per group were required (38.0° vs. 31.9°, SD = 5°), and 16 participants for the downward phase (41.2° vs. 36.2°, SD = 5°). As these calculations were based on comparisons between two groups, the largest estimate corresponded to 32 participants in total. A minimum of 36 participants was considered appropriate to allow for potential data loss due to technical issues or marker occlusion during motion capture.

### Recruitment

Participants were recruited between April and July 2025 from five primary care clinics in Northern Sweden and through public advertisements. Study procedures, interventions, and data collection were conducted between September and October 2025. Clinics received written information on the study’s background, design, procedures, interventions, time commitment, and eligibility criteria, which they used to identify potentially eligible participants and provide study information. Individuals responding to advertisements were screened for eligibility by a licensed chiropractor, who confirmed eligibility prior to inclusion. All potentially eligible participants received detailed study information (Additional file 1) covering the study purpose, procedures, interventions, potential risks and benefits, data handling, and participants’ rights. After reviewing this information and completing the eligibility assessment, participants were able to ask questions before deciding whether to enroll. No financial or other incentives were provided.

### Randomization

Participants were randomized to one of six intervention orders and one of 24 measurement sequences (Additional file 2) using an online research randomizer [31]. The randomization sequence was generated by the last author prior to participant inclusion and concealed until enrollment. The 24 sequences were pre-arranged in blocks to keep each pair of lift, gait, and forward flexion tests together while still varying the order within each block. Participants and the treating chiropractor were aware of the intervention being delivered. Further details are provided in Additional file 2.

### Lifting procedure

**Fig. 1:**
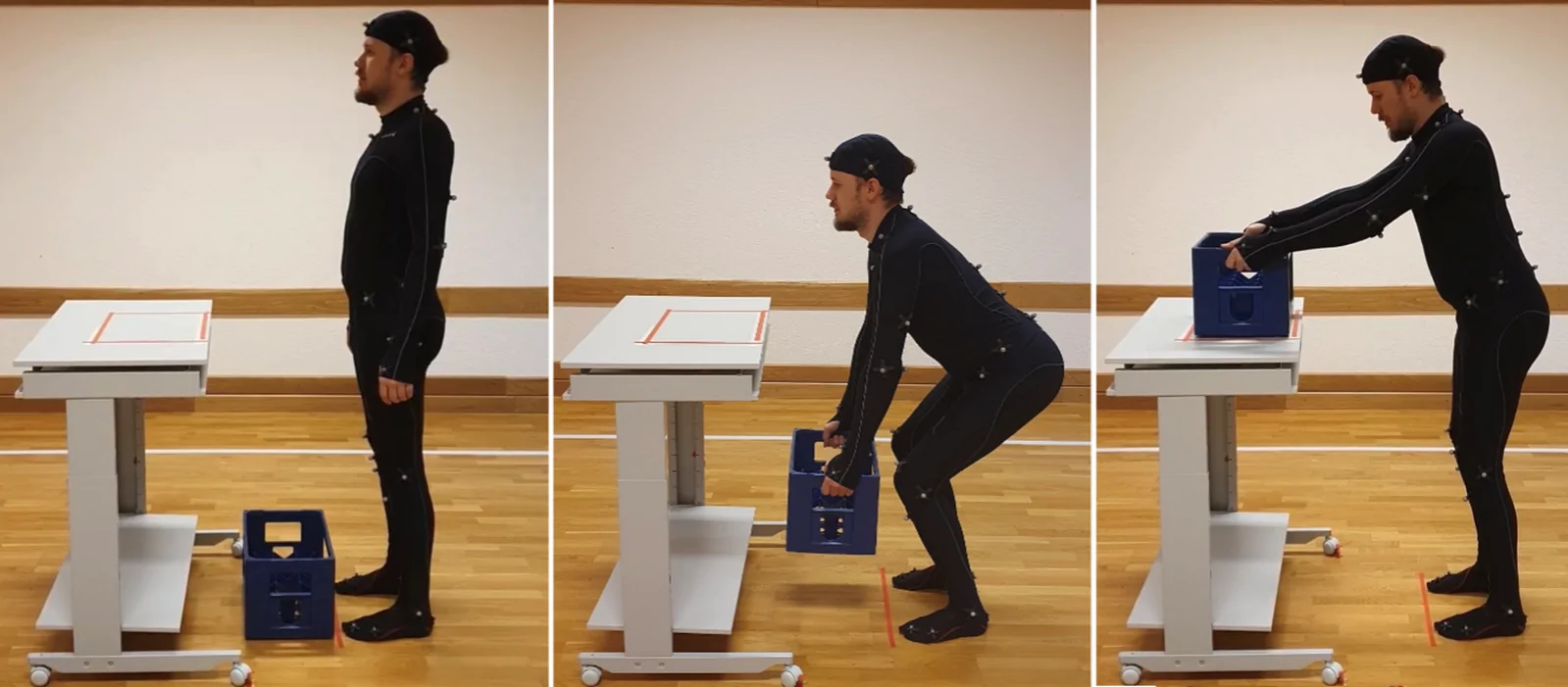
Set-up for the lifting task. Participants lifted a plastic box with light and heavy loads from the marked area on the floor to the table using a symmetrical lift performed with both hands

Participants lifted a plastic box (0.495 kg; 30 × 40 × 30 cm) from the floor to a table (Fig. 1). No instructions were provided regarding lifting posture or technique, and participants were allowed to perform the lift in their preferred manner. Participants selected a weight they considered light (2–5 kg) and one they considered heavy (6–15 kg), with the heavy load three times the light load. Table height was set to 50% of body height and distance to 75% of arm length, with tape markings standardizing start and end positions. The test leader returned the box between lifts to minimize fatigue.

### Motion-capture setup

Movement characteristics data were collected using a motion capture technique (Qualisys Track Manager (QTM)) together with the Calqulus analysis module [32]. The system consisted of 16 Qualisys Miqus M3 infrared cameras operating at 120 Hz and covering a capture volume of approximately 6 × 5 × 3 m. Participants wore a tight-fitting marker suit with 41 reflective markers placed according to the Qualisys sports marker set [33], enabling three-dimensional full-body kinematic analysis. Additional markers were placed at Th7 vertebra, at the fingertip of each middle finger, and on the box to enable detailed in-depth motion analysis of different parts of the trunk, the forward flexion tasks and for identification of lifting phases, respectively, totaling 45 reflective markers.

#### Outcome measures

The primary outcomes were objective movement characteristics of the lifting tasks derived from three-dimensional motion capture data. The primary endpoint was the immediate post-intervention assessment at T1. Secondary outcomes included pain intensity, perceived effectiveness, willingness to undergo the interventions, and treatment preference.

### Kinematic variables of movement performance

Movement characteristics were derived from three-dimensional motion capture data collected at T0, T1, T2, and T3 to characterize lifting performance across temporal, spatial, and spatiotemporal domains. Temporal outcomes included movement time and phase durations. Spatial outcomes included ROM and box displacement. Spatiotemporal outcomes included movement speed, movement smoothness (spectral arc length, SPARC), and intersegmental coordination between back and knee movement during the lifting task.

### PROMs

Self-reported outcomes included pain intensity, perceived effectiveness, willingness to undergo the interventions, and treatment preference. Pain intensity was assessed at T0, T1, T2, and T3 using an 11-point numerical rating scale (NRS), where participants rated their pain “right now” from 0 (“no pain”) to 10 (“worst imaginable pain”). Perceived effectiveness and willingness to undergo each intervention were assessed at T3 using 11-point scales ranging from 0 to 10, with higher scores indicating greater perceived effectiveness and willingness to undergo the intervention. Treatment preference was assessed at T3 by asking participants to rank the three interventions from most preferred (#1) to least preferred (#3), with each intervention assigned a unique rank to establish a clear hierarchy of preferences. Further details on PROMs (Additional file 3).

### Interventions

#### SMT

**Fig. 2:**
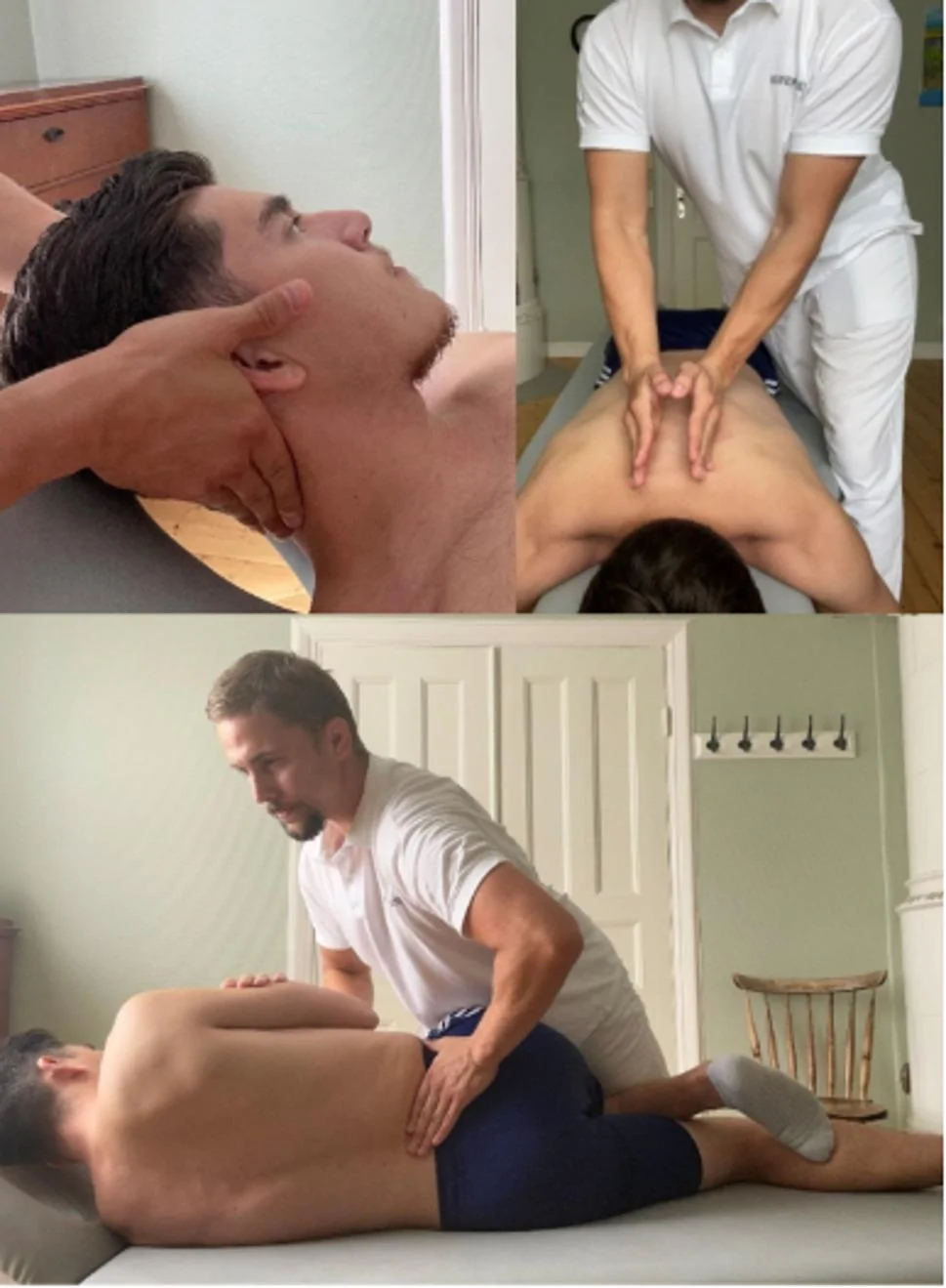
Examples of HVLA spinal manipulation of the cervical, thoracic, and lumbar spine

A licensed chiropractor performed a standardized examination of the spine and pelvis before manually applying SMT to spinal segments and related joints assessed to have restricted ROM. Motion palpation was used to identify target motion segments. Participants were positioned in supine, prone, or side-lying positions depending on the spinal region treated (Fig. 2). SMT was delivered using a high-velocity, low-amplitude (HVLA) thrust technique applied beyond passive ROM, without provoking pain. Details on treatment dose and treated spinal segments (Additional file 4).

#### ET

**Fig. 3:**
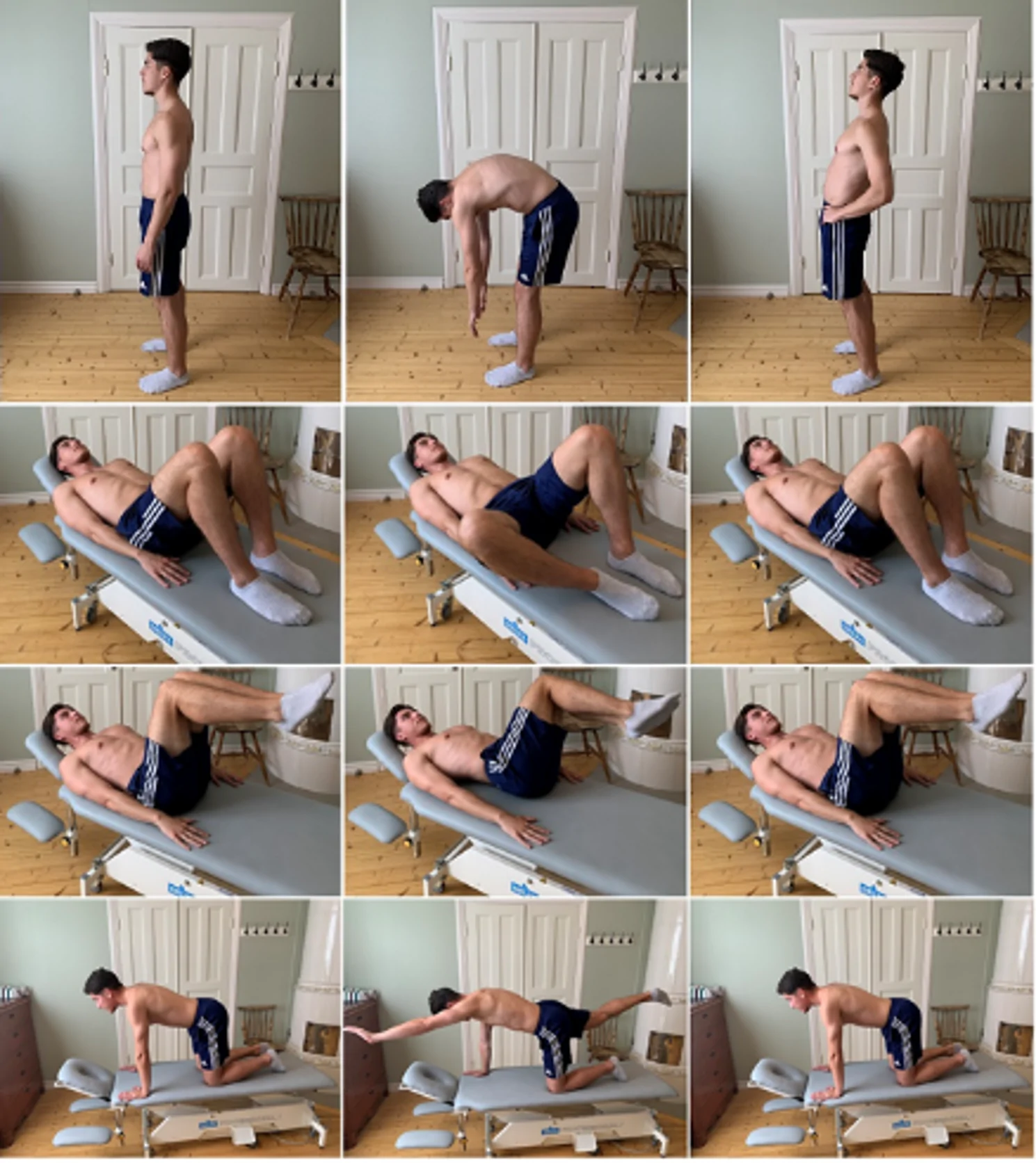
Exercise therapy exercises: (1) forward bending; (2 A) leg fall-out, easier version; (2B) leg fall-out, more challenging version; and (3) bird-dog

The exercise therapy intervention consisted of three motor control exercises performed in different movement directions, targeting trunk, hip, and pelvic control (Fig. 3). Exercises were performed under the supervision of a licensed chiropractor, within a pain-free ROM, and in a controlled manner. The first exercise, forward bending, was performed for three repetitions. The other two exercises were performed for three repetitions per side with a 5-second hold; for the second exercise, participants could choose between an easier or a more challenging variation. Fewer repetitions were permitted if pain occurred. Details on exercise dose and exercises performed (Additional file 5).

#### OLP

**Fig. 4:**
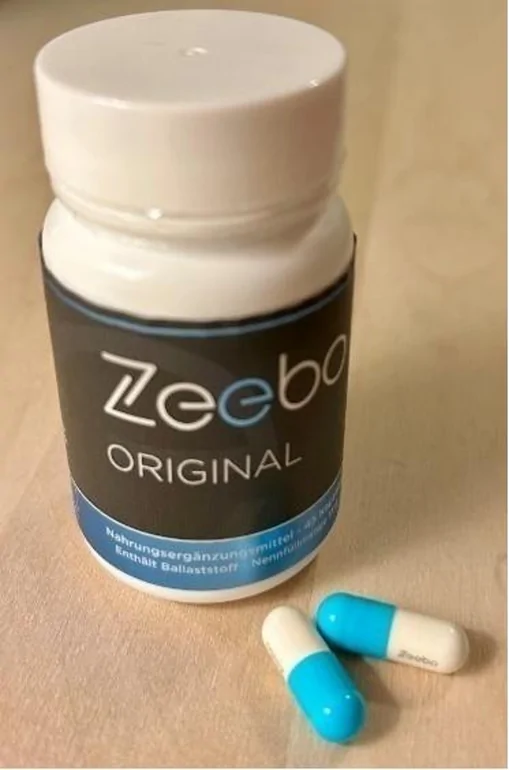
Bottle and placebo capsules containing microcrystalline cellulose

Participants received standardized information about placebo effects through an information video (Additional file 6) and a brief verbal explanation, both based on the procedure described by Carvalho et al. [34]. The information conveyed that placebo effects can be potent, are supported by previous research, and may occur even without a positive attitude toward the treatment. The example of Pavlovian conditioning was included as a simple illustration of how placebo effects may occur. Participants then ingested, under supervision, two open-label placebo capsules clearly labeled as “placebo” (Fig. 4), containing the inert substance microcrystalline cellulose.

### Data analysis

#### Temporal outcomes

Total movement time was defined as the time interval between the start of the downward phase and the end of the upward phase of the lifting movement. The start of the downward phase was defined as the first frame before the marker on the Th2 vertebra moved downward for 24 consecutive frames, and the start of the upward phase as the first frame before the box marker moved upward for 24 consecutive frames. The end of the movement was defined as the frame where the box marker, after reaching its maximum height, stopped decreasing along the Z-axis (vertical). The box was considered stationary if its Z-position varied by no more than ± 1 mm for 12 consecutive frames.

#### Spatial outcomes

Box displacement was calculated as the two-dimensional distance traveled in the sagittal (X–Z) plane (anterior–posterior, vertical) using the Pythagorean theorem, from the start of the upward phase to the end of the movement, and was expressed in millimeters (mm). Thoracic flexion was calculated from markers on Th2, Th7, and Th12 in the sagittal (X–Z) plane from the start of the downward phase to the end of the movement, and the maximum value during this interval was used for analysis. Thorax–pelvis tilt, representing the relative sagittal-plane movement between the thorax and pelvis, was obtained directly from the Calqulus analysis module (Qualisys), and the maximum value during each lifting task was used as a proxy for maximum lumbar flexion. The maximum hip and knee flexion values were obtained directly from the Calqulus analysis module reports. All angular measures were expressed in degrees (°).

#### Spatiotemporal outcomes

Movement speed was calculated as the mean speed of the box during the upward phase, defined as the two-dimensional distance traveled in the sagittal (X–Z) plane divided by the time interval from the start of the upward phase to the end of the movement.

Movement smoothness was quantified using Spectral Arc Length (SPARC), a robust and validated measure derived from the frequency-domain representation of velocity [35]. SPARC was calculated from the resultant velocity profiles of markers placed on vertebral levels Th2, Th7, Th12, and the waist during the upward phase of the lift. Velocity signals were transformed to the frequency domain using a Fourier transform and normalized to the maximum spectral amplitude. A cut-off frequency of 10 Hz was applied, as the lifting task represents a voluntary, goal-directed movement where the majority of relevant signal content is expected below this range, while higher frequencies are more likely to reflect noise rather than meaningful movement characteristics. An amplitude threshold of 0.05 was used to identify the relevant frequency components contributing to movement smoothness, consistent with previous implementations of the SPARC method [36]. SPARC was computed as the arc length of the normalized spectrum up to 10 Hz. SPARC is a unitless measure with values negative by convention, where values closer to zero indicate smoother movement.

The back-to-knee movement was analyzed using functional data analysis (FDA), a statistical framework for analyzing continuous time-series data [37]. Such approaches have been advocated for biomechanical trajectory analysis to avoid limitations associated with discrete, zero-dimensional analyses [38]. This outcome reflects the relative contribution of sagittal-plane motion (i.e., flexion/extension) of the back and knees during the lifting cycle. Back flexion was calculated from markers on Th2, Th7, and the waist in the sagittal (X–Z) plane. Changes in back and knee angles relative to the start of the lifting cycle were calculated, and the difference between these changes was used to characterize the relative contribution of back versus knee movement. Raw marker trajectories were imported into R (version 4.5.2) [39], and continuous time-series data were processed using the FDA package [40]. Time-normalized kinematic waveforms were modeled as continuous functions over the interval 0–1 to account for temporal dependencies across the lifting cycle. Functional data were represented using B-spline basis functions (*n* = 12), and individual trajectories were retained for each participant. Difference curves were computed by subtracting baseline from follow-up trajectories, and group mean difference curves were calculated to summarize overall changes and identify time intervals with differing movement strategies. Positive values indicate greater relative movement in the back (back dominance), whereas negative values indicate greater movement in the knees (knee dominance). The average onset of the upward phase was used to indicate the start of lifting and is marked by vertical lines (Figs. 6 and 7). Trajectories were visually inspected prior to analysis, and participants with non-physiological kinematic patterns likely due to marker occlusion were excluded (*n* = 4): one from each of the SMT and ET groups during the light lift, and one from each of the SMT and OLP groups during the heavy lift.

#### Statistical analysis

All analyses were conducted in R (version 4.5.2). Statistical significance was set at *p* < 0.05 throughout. Continuous outcomes were analyzed using linear mixed-effects models to account for repeated measurements within participants over time. Models included group, time, and the group × time interaction as fixed effects, with participant ID included as a random effect. Pairwise comparisons were performed using estimated marginal means with Tukey adjustment for multiple comparisons to examine between-group differences at T0 and T1 and within-group changes from T0 to T1. Analyses were performed according to the intention-to-treat principle. All randomized participants were included in all analyses, except for FDA trajectories excluded due to substantial artefacts or marker occlusion.

Secondary analyses of continuous outcomes were conducted to evaluate the add-on effects of OLP. Change scores were compared between participants receiving SMT + ET alone (T0 to T1) and those receiving SMT + ET followed by OLP (T0 to T2). These comparisons were performed using a linear model with cluster-robust standard errors, clustered by participant ID to account for individuals contributing data to both groups.

Differences in perceived effectiveness, willingness to undergo the interventions, and intervention rankings at T3 were analyzed using Friedman tests, followed by post hoc pairwise comparisons with Wilcoxon signed-rank tests and Bonferroni correction. Cronbach’s alpha was calculated to assess the internal consistency of the PROMs assessing perceived effectiveness, willingness to undergo the interventions, and intervention rankings.

For FDA of kinematic trajectories, permutation-based tests were used to assess differences across the time-normalized domain. Statistical inference was based on an adjusted p-value function controlling the family-wise error rate across the continuous time series. Statistically significant time intervals were identified to determine when the relative contributions of back and knee motion differed significantly. For group comparisons at T1, differences in mean functional trajectories were assessed using permutation tests.

## Results

**Fig. 5:**
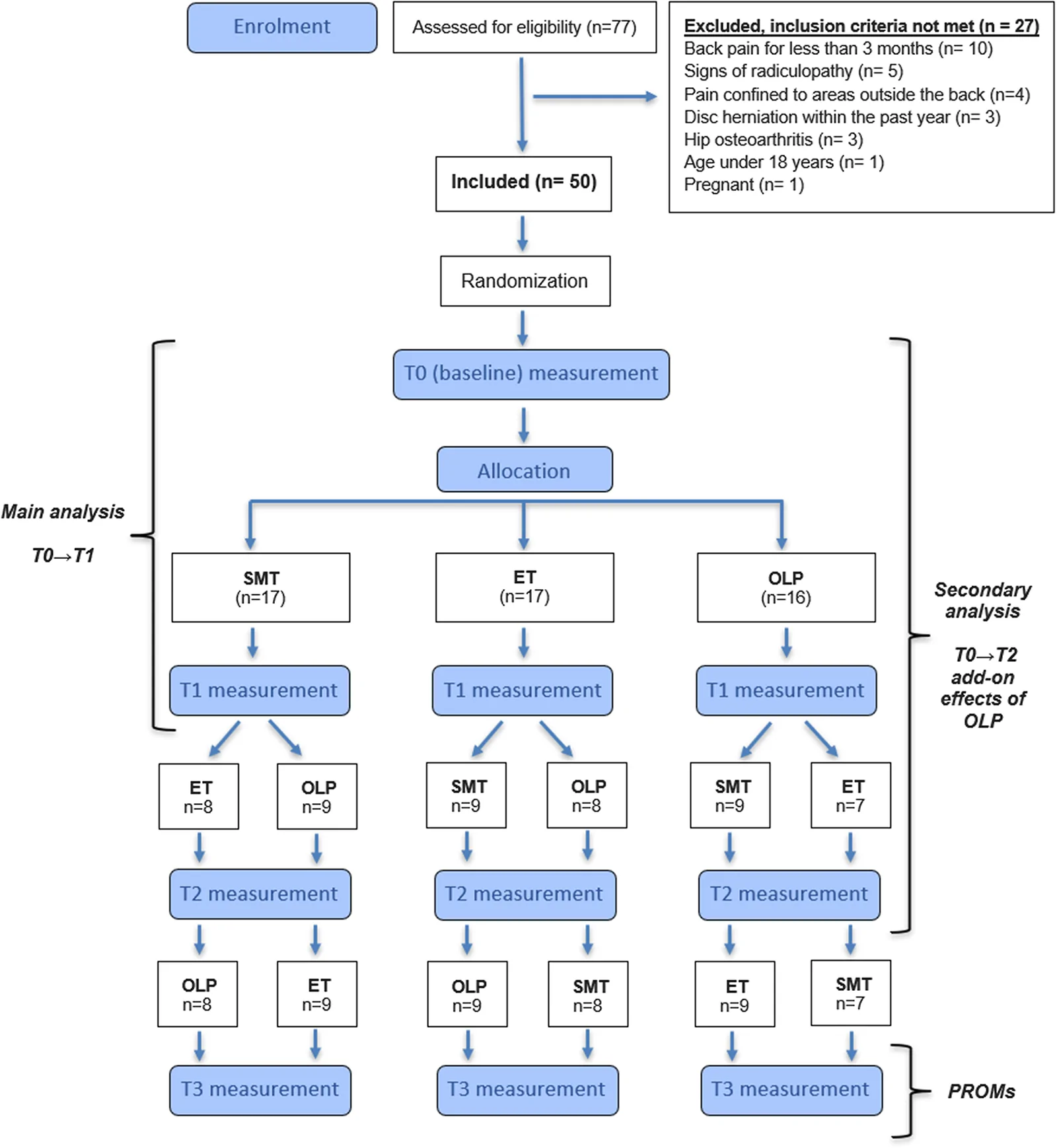
CONSORT flow chart

Descriptive statistics for participant characteristics are presented in Table 1. A total of 77 individuals were assessed for eligibility; 27 were excluded for not meeting the inclusion criteria. The remaining 50 participants were randomized and allocated to three groups: SMT (*n* = 17), ET (*n* = 17), and OLP (*n* = 16)(Fig. 5). All participants underwent baseline measurements (T0) before receiving the assigned intervention. The study employed a crossover design in which the order of interventions was determined during the initial randomization, ensuring that all participants eventually received SMT, ET, and OLP. Follow-up measurements (T1, T2, T3) were conducted after each intervention, allowing both primary and secondary analyses of the effects of the interventions. At T3, participants also ranked the three interventions according to their treatment preference.

All interventions were delivered as intended, and all participants completed the assigned intervention sessions. Adverse events were systematically assessed, and no serious adverse events occurred during the trial.

**Table 1:** Baseline characteristics of study participants

Characteristics	All (*n* = 50)	SMT (*n* = 17)	ET (*n* = 17)	OLP (*n* = 16)
Gender (female/male/other)	25/24/1	5/12/0	7/9/1	13/3/0
Age (years), mean ± SD	49.3 ± 14.2	46.8 ± 16.3	50.4 ± 14.3	50.6 ± 12.1
BMI (kg/m²), mean ± SD	26.7 ± 4.7	27.8 ± 5.0	26.5 ± 5.5	25.7 ± 3.5
Education level (elementary/high school/university)	0/16/34	0/8/9	0/4/13	0/4/12
Employment status (employed/unemployed/student/retired)	40/0/3/7	14/0/1/2	13/0/1/3	13/0/1/2
Physical activity guideline adherence* (yes/no/don’t know)	37/11/2	10/6/1	13/4/0	14/1/1
Back pain duration > 3 months, n (%)	50 (100)	17 (100)	17 (100)	16 (100)
Sought care for back pain, n (%)	50 (100)	17 (100)	17 (100)	16 (100)
ODI (%, disability), mean ± SD	19.6 ± 11.1	16.5 ± 9.4	20.2 ± 12.3	22.3 ± 11.4
Pain intensity (NRS, baseline), mean ± SD	3.0 ± 1.8	3.7 ± 1.8	2.6 ± 1.7	2.8 ± 1.7
Other medical condition causing pain, n (%)	0 (0)	0 (0)	0 (0)	0 (0)
Medication use past week (none/NSAID/opioid/non-opioid analgesics)	23/15/1/20	7/6/0/8	8/4/1/6	8/5/0/6

Values are presented as mean ± standard deviation (SD) for normally distributed data. *Self-reported adherence to WHO physical activity guidelines. ODI: Oswestry Disability Index; NRS: Numeric Rating Scale

For pain intensity, no significant between-group differences were observed at baseline (T0) or at the primary endpoint (T1) (all *p* ≥ 0.15; Table 2a). A significant within-group reduction in pain from T0 to T1 was observed in the SMT group (Δ = 1.12, *p* < 0.001), whereas no significant changes were observed in the ET or OLP groups. In the add-on analysis, no significant difference in change was observed between treatments with and without OLP. The reduction in pain was similar between SMT/ET alone (T0–T1) and SMT/ET combined with OLP (T0–T2) (Δ = 0.02, *p* = 0.91).

### Patient-reported outcome measures

**Table 2:** Changes in pain intensity

Outcome / Analysis	Comparison	Δ	95% CI	*p*-value
Pain				
Between-group (T0)	SMT vs. ET	1.06	[-0.29, 2.40]	0.150
	SMT vs. OLP	0.90	[-0.47, 2.26]	0.264
	ET vs. OLP	-0.16	[-1.53, 1.20]	0.957
Between-group (T1)	SMT vs. ET	0.35	[-0.99, 1.70]	0.805
	SMT vs. OLP	0.09	[-1.27, 1.46]	0.986
	ET vs. OLP	-0.26	[-1.63, 1.11]	0.891
Within-group (T0–T1)	SMT	1.12	[0.46, 1.77]	**< 0.001**
	SMT*	1.19	[0.66, 1.71]	**< 0.001**
	ET	0.41	[-0.24, 1.07]	0.363
	ET*	0.47	[-0.08, 1.01]	0.092
	OLP	0.31	[-0.36, 0.99]	0.626
	OLP*	0.36	[-0.21, 0.92]	0.212
Add-on (OLP effect)	(T0–T2) vs. (T0–T1)	0.02	[-0.36, 0.40]	0.905

Values represent estimated mean differences (Δ) with 95% confidence intervals derived from linear mixed-effects models with Tukey-adjusted pairwise comparisons. The add-on analysis compares change from T0–T2 (SMT/ET + OLP) with T0–T1 (SMT/ET alone). *Sensitivity analysis excluding participants reporting no pain at baseline (NRS = 0)

SMT received the highest ratings for both perceived effectiveness and willingness to undergo the intervention, followed by ET, whereas OLP received the lowest ratings (Table 2b). Post hoc analyses showed significant differences between all intervention pairs for both effectiveness and willingness (all *p* < 0.05). For ranking, OLP was ranked lower than both SMT and ET (both *p* < 0.001), whereas no statistically significant difference was observed between SMT and ET (*p* = 0.068). Internal consistency across the measures was low (Cronbach’s α = 0.45, 95% CI 0.19–0.66), with a low average inter-item correlation (*r* = 0.03), indicating that the outcomes capture related but distinct aspects of participants’ evaluations.

**Table 3:** Patient evaluations at T3

Outcome	SMT	ET	OLP	Test statistic	Kendall’s W	*p*-value
Perceived effectiveness, mean ± SD	7.0 ± 2.1	6.2 ± 2.3	2.2 ± 2.7	χ²(2) = 58.2	0.582	< 0.001
Willingness to undergo the intervention, mean ± SD	9.1 ± 1.5	8.2 ± 2.1	4.7 ± 3.8	χ²(2) = 42.4	0.424	< 0.001
Intervention ranking, mean rank	1.36	1.72	2.92	χ²(2) = 66.7	0.667	< 0.001

Values are based on Friedman tests with Kendall’s W as effect size. Post hoc pairwise comparisons were performed using Wilcoxon signed-rank tests with Bonferroni correction

Temporal outcomes remained similar across interventions in both the light and heavy lift tasks (Table 3). Neither movement time nor phase durations changed significantly over time or differed between groups. An add-on effect of OLP was observed for the upward phase during the heavy lift, while all other temporal measures showed no significant changes.

### Temporal outcome measures

**Table 4:** Total movement time and phase durations

Outcome / Analysis	Comparison	Δ	95% CI	*p*-value
**Light lift total time (s)**				
Between-group (T0)	SMT vs. ET	0.09	[-0.49, 0.68]	0.925
	SMT vs. OLP	-0.07	[-0.67, 0.52]	0.953
	ET vs. OLP	-0.17	[-0.76, 0.43]	0.783
Between-group (T1)	SMT vs. ET	0.35	[-0.24, 0.93]	0.337
	SMT vs. OLP	-0.17	[-0.77, 0.42]	0.767
	ET vs. OLP	-0.52	[-1.11, 0.07]	0.098
Within-group (T0–T1)	SMT	0.08	[-0.32, 0.48]	0.890
	ET	0.33	[-0.07, 0.73]	0.120
	OLP	-0.02	[-0.43, 0.39]	0.990
Add-on (OLP effect)	(T0–T2) vs. (T0–T1)	-0.07	[-0.44, 0.29]	0.690
**Light lift down phase (s)**				
Between-group (T0)	SMT vs. ET	0.04	[-0.28, 0.36]	0.956
	SMT vs. OLP	-0.02	[-0.35, 0.30]	0.987
	ET vs. OLP	-0.06	[-0.38, 0.27]	0.901
Between-group (T1)	SMT vs. ET	0.18	[-0.14, 0.50]	0.370
	SMT vs. OLP	-0.02	[-0.34, 0.30]	0.988
	ET vs. OLP	-0.20	[-0.53, 0.12]	0.304
Within-group (T0–T1)	SMT	0.02	[-0.24, 0.28]	0.980
	ET	0.16	[-0.10, 0.42]	0.290
	OLP	0.02	[-0.25, 0.29]	0.980
Add-on (OLP effect)	(T0–T2) vs. (T0–T1)	-0.09	[-0.31, 0.14]	0.470
**Light lift upward phase (s)**				
Between-group (T0)	SMT vs. ET	0.05	[-0.29, 0.40]	0.925
	SMT vs. OLP	-0.05	[-0.40, 0.29]	0.931
	ET vs. OLP	-0.11	[-0.45, 0.24]	0.746
Between-group (T1)	SMT vs. ET	0.16	[-0.18, 0.51]	0.486
	SMT vs. OLP	-0.15	[-0.50, 0.19]	0.549
	ET vs. OLP	-0.32	[-0.66, 0.03]	0.081
Within-group (T0–T1)	SMT	0.06	[-0.16, 0.28]	0.810
	ET	0.17	[-0.05, 0.39]	0.170
	OLP	-0.04	[-0.27, 0.18]	0.890
Add-on (OLP effect)	(T0–T2) vs. (T0–T1)	0.01	[-0.17, 0.19]	0.910
**Heavy lift total time (s)**				
Between-group (T0)	SMT vs. ET	0.22	[-0.50, 0.93]	0.751
	SMT vs. OLP	-0.41	[-1.13, 0.32]	0.375
	ET vs. OLP	-0.63	[-1.35, 0.10]	0.105
Between-group (T1)	SMT vs. ET	-0.01	[-0.73, 0.70]	0.999
	SMT vs. OLP	-0.32	[-1.05, 0.41]	0.548
	ET vs. OLP	-0.31	[-1.03, 0.42]	0.574
Within-group (T0–T1)	SMT	0.16	[-0.31, 0.63]	0.680
	ET	-0.07	[-0.53, 0.40]	0.940
	OLP	0.25	[-0.23, 0.74]	0.430
Add-on (OLP effect)	(T0–T2) vs. (T0–T1)	0.13	[-0.19, 0.45]	0.430
**Heavy lift down phase (s)**				
Between-group (T0)	SMT vs. ET	0.07	[-0.40, 0.53]	0.939
	SMT vs. OLP	-0.17	[-0.63, 0.30]	0.679
	ET vs. OLP	-0.23	[-0.70, 0.24]	0.471
Between-group (T1)	SMT vs. ET	0.03	[-0.44, 0.49]	0.990
	SMT vs. OLP	-0.11	[-0.58, 0.36]	0.846
	ET vs. OLP	-0.14	[-0.60, 0.33]	0.772
Within-group (T0–T1)	SMT	-0.01	[-0.36, 0.34]	1.00
	ET	-0.05	[-0.40, 0.30]	0.930
	OLP	0.04	[-0.32, 0.40]	0.960
Add-on (OLP effect)	(T0–T2) vs. (T0–T1)	-0.06	[-0.31, 0.19]	0.660
**Heavy lift upward phase (s)**				
Between-group (T0)	SMT vs. ET	0.15	[-0.25, 0.56]	0.648
	SMT vs. OLP	-0.24	[-0.65, 0.17]	0.340
	ET vs. OLP	-0.39	[-0.81, 0.02]	0.063
Between-group (T1)	SMT vs. ET	-0.04	[-0.44, 0.37]	0.971
	SMT vs. OLP	-0.21	[-0.62, 0.20]	0.443
	ET vs. OLP	-0.17	[-0.58, 0.24]	0.582
Within-group (T0–T1)	SMT	0.18	[-0.07, 0.42]	0.200
	ET	-0.01	[-0.26, 0.23]	0.990
	OLP	0.21	[-0.04, 0.46]	0.120
Add-on (OLP effect)	(T0–T2) vs. (T0–T1)	0.19	[0.02, 0.35]	**0.035**

Mean differences (Δ) are presented with 95% confidence intervals (CI). Between- and within-group comparisons were estimated using linear mixed models with Tukey adjustment. Within-group changes represent T0–T1 differences. The add-on analysis compared change in SMT/ET + OLP (T0–T2) with SMT/ET (T0–T1) using a linear model with cluster-robust standard errors clustered on participant ID. Bold p-values indicate *p* < 0.05

Spatial outcomes showed a small number of significant within-group changes (Table 4). SMT was associated with reduced thorax–pelvis tilt (a proxy for lumbar flexion) and increased hip flexion during the heavy lift, while OLP was associated with reduced thorax–pelvis tilt and increased hip and knee flexion during the light lift. OLP also demonstrated add-on effects for hip flexion in both lifting conditions. Aside from these findings, most spatial measures remained stable and no significant between-group differences were identified.

### Spatial outcome measures

**Table 5:** Box displacement, thoracic flexion, thorax-pelvis tilt, hip flexion, and knee flexion

Outcome / Analysis	Comparison	Δ	95% CI	*p*-value
**Light lift box displacement (mm)**				
Between-group (T0)	SMT vs. ET	-0.56	[-76.94, 75.83]	1.000
	SMT vs. OLP	-0.97	[-78.54, 76.60]	1.000
	ET vs. OLP	-0.42	[-77.98, 77.15]	1.000
Between-group (T1)	SMT vs. ET	36.50	[-39.88, 112.88]	0.493
	SMT vs. OLP	-5.11	[-82.67, 72.46]	0.987
	ET vs. OLP	-41.61	[-119.17, 35.96]	0.411
Within-group (T0–T1)	SMT	-21.12	[-74.51, 32.27]	0.615
	ET	15.94	[-37.45, 69.32]	0.758
	OLP	-25.26	[-80.29, 29.78]	0.521
Add-on (OLP effect)	(T0–T2) vs. (T0–T1)	38.59	[-3.22, 80.40]	0.075
**Heavy lift box displacement (mm)**				
Between-group (T0)	SMT vs. ET	28.96	[-74.61, 132.53]	0.782
	SMT vs. OLP	-9.25	[-114.43, 95.92]	0.976
	ET vs. OLP	-38.22	[-143.39, 66.96]	0.661
Between-group (T1)	SMT vs. ET	9.75	[-93.82, 113.32]	0.972
	SMT vs. OLP	2.15	[-103.02, 107.33]	0.999
	ET vs. OLP	-7.59	[-112.77, 97.58]	0.984
Within-group (T0–T1)	SMT	14.79	[-44.28, 73.86]	0.823
	ET	-4.43	[-63.50, 54.64]	0.983
	OLP	26.19	[-34.69, 87.08]	0.563
Add-on (OLP effect)	(T0–T2) vs. (T0–T1)	41.83	[-4.76, 88.43]	0.083
**Light lift thoracic flexion (°)**				
Between-group (T0)	SMT vs. ET	0.93	[-3.06, 4.91]	0.840
	SMT vs. OLP	-2.37	[-6.41, 1.68]	0.341
	ET vs. OLP	-3.30	[-7.34, 0.75]	0.131
Between-group (T1)	SMT vs. ET	1.17	[-2.82, 5.15]	0.760
	SMT vs. OLP	-2.70	[-6.75, 1.35]	0.250
	ET vs. OLP	-3.87	[-7.91, 0.18]	0.064
Within-group (T0–T1)	SMT	0.32	[-0.38, 1.03]	0.526
	ET	0.56	[-0.14, 1.27]	0.147
	OLP	-0.01	[-0.74, 0.72]	0.999
Add-on (OLP effect)	(T0–T2) vs. (T0–T1)	0.18	[-0.28, 0.63]	0.453
**Heavy lift thoracic flexion (°)**				
Between-group (T0)	SMT vs. ET	0.70	[-3.25, 4.65]	0.904
	SMT vs. OLP	-2.71	[-6.72, 1.29]	0.240
	ET vs. OLP	-3.41	[-7.42, 0.59]	0.109
Between-group (T1)	SMT vs. ET	1.04	[-2.91, 4.99]	0.800
	SMT vs. OLP	-2.27	[-6.28, 1.73]	0.364
	ET vs. OLP	-3.31	[-7.32, 0.69]	0.123
Within-group (T0–T1)	SMT	0.26	[-0.53, 1.05]	0.722
	ET	0.60	[-0.19, 1.39]	0.176
	OLP	0.70	[-0.12, 1.51]	0.109
Add-on (OLP effect)	(T0–T2) vs. (T0–T1)	-0.13	[-0.69, 0.43]	0.647
**Light lift thorax–pelvis tilt (°)**				
Between-group (T0)	SMT vs. ET	1.36	[-7.73, 10.45]	0.931
	SMT vs. OLP	-0.38	[-9.61, 8.85]	0.995
	ET vs. OLP	-1.74	[-10.97, 7.50]	0.893
Between-group (T1)	SMT vs. ET	5.91	[-3.18, 15.00]	0.269
	SMT vs. OLP	-1.72	[-10.96, 7.51]	0.895
	ET vs. OLP	-7.64	[-16.87, 1.60]	0.124
Within-group (T0–T1)	SMT	-2.84	[-5.75, 0.08]	0.059
	ET	1.72	[-1.20, 4.63]	0.344
	OLP	-4.18	[-7.19, -1.17]	**0.004**
Add-on (OLP effect)	(T0–T2) vs. (T0–T1)	-1.35	[-3.43, 0.73]	0.206
**Heavy lift thorax–pelvis tilt (°)**				
Between-group (T0)	SMT vs. ET	1.79	[-7.18, 10.76]	0.880
	SMT vs. OLP	-0.75	[-9.86, 8.35]	0.978
	ET vs. OLP	-2.54	[-11.65, 6.56]	0.780
Between-group (T1)	SMT vs. ET	5.51	[-3.46, 14.48]	0.307
	SMT vs. OLP	1.21	[-7.89, 10.32]	0.945
	ET vs. OLP	-4.30	[-13.41, 4.81]	0.494
Within-group (T0–T1)	SMT	-3.53	[-5.83, -1.23]	**0.001**
	ET	0.19	[-2.10, 2.49]	0.978
	OLP	-1.56	[-3.93, 0.80]	0.263
Add-on (OLP effect)	(T0–T2) vs. (T0–T1)	-1.05	[-2.86, 0.75]	0.258
**Light lift hip flexion (°)**				
Between-group (T0)	SMT vs. ET	-3.06	[-12.91, 6.80]	0.737
	SMT vs. OLP	-1.58	[-11.59, 8.43]	0.924
	ET vs. OLP	1.48	[-8.53, 11.49]	0.933
Between-group (T1)	SMT vs. ET	-5.90	[-15.76, 3.95]	0.326
	SMT vs. OLP	2.47	[-7.54, 12.48]	0.824
	ET vs. OLP	8.37	[-1.64, 18.38]	0.118
Within-group (T0–T1)	SMT	1.52	[-1.53, 4.57]	0.465
	ET	-1.33	[-4.38, 1.73]	0.557
	OLP	5.57	[2.42, 8.72]	**< 0.001**
Add-on (OLP effect)	(T0–T2) vs. (T0–T1)	2.94	[1.09, 4.78]	**0.003**
**Heavy lift hip flexion (°)**				
Between-group (T0)	SMT vs. ET	-3.50	[-13.90, 6.90]	0.697
	SMT vs. OLP	-1.98	[-12.54, 8.58]	0.894
	ET vs. OLP	1.53	[-9.03, 12.08]	0.935
Between-group (T1)	SMT vs. ET	-7.24	[-17.64, 3.15]	0.222
	SMT vs. OLP	-3.24	[-13.80, 7.32]	0.741
	ET vs. OLP	4.00	[-6.56, 14.56]	0.634
Within-group (T0–T1)	SMT	3.28	[0.31, 6.24]	**0.026**
	ET	-0.46	[-3.43, 2.50]	0.926
	OLP	2.01	[-1.04, 5.07]	0.264
Add-on (OLP effect)	(T0–T2) vs. (T0–T1)	2.92	[1.04, 4.80]	**0.003**
**Light lift knee flexion (°)**				
Between-group (T0)	SMT vs. ET	-4.50	[-20.00, 11.00]	0.766
	SMT vs. OLP	-13.32	[-29.05, 2.42]	0.113
	ET vs. OLP	-8.81	[-24.55, 6.92]	0.377
Between-group (T1)	SMT vs. ET	-7.94	[-23.44, 7.55]	0.441
	SMT vs. OLP	-4.74	[-20.47, 11.00]	0.752
	ET vs. OLP	3.21	[-12.53, 18.95]	0.877
Within-group (T0–T1)	SMT	2.03	[-5.70, 9.76]	0.806
	ET	-1.41	[-9.14, 6.32]	0.902
	OLP	10.61	[2.64, 18.58]	**0.006**
Add-on (OLP effect)	(T0–T2) vs. (T0–T1)	2.94	[-1.94, 7.81]	0.242
**Heavy lift knee flexion (°)**				
Between-group (T0)	SMT vs. ET	-7.51	[-23.29, 8.27]	0.491
	SMT vs. OLP	-11.29	[-27.32, 4.74]	0.216
	ET vs. OLP	-3.78	[-19.81, 12.25]	0.838
Between-group (T1)	SMT vs. ET	-6.48	[-22.26, 9.30]	0.588
	SMT vs. OLP	-7.51	[-23.54, 8.51]	0.501
	ET vs. OLP	-1.03	[-17.06, 14.99]	0.987
Within-group (T0–T1)	SMT	1.29	[-5.09, 7.66]	0.881
	ET	2.32	[-4.06, 8.69]	0.663
	OLP	5.06	[-1.51, 11.64]	0.164
Add-on (OLP effect)	(T0–T2) vs. (T0–T1)	1.94	[-2.99, 6.88]	0.443

Mean differences (Δ) are presented with 95% confidence intervals (CI). Between- and within-group comparisons were estimated using linear mixed models with Tukey adjustment. Within-group changes represent T0–T1 differences. The add-on analysis compared change in SMT/ET + OLP (T0–T2) with SMT/ET (T0–T1) using a linear model with cluster-robust standard errors clustered on participant ID. Bold p-values indicate *p* < 0.05

Spatiotemporal outcomes were consistent across interventions (Table 5). Movement smoothness (SPARC) and movement speed showed no significant changes over time and did not differ significantly between groups in either lifting condition, indicating stable movement quality.

### Spatiotemporal outcome measures

**Table 6:** SPARC, movement speed

Outcome / Analysis	Comparison	Δ	95% CI	*p*-value
**Light lift SPARC**				
Between-group (T0)	SMT vs. ET	-0.03	[-0.18, 0.11]	0.842
	SMT vs. OLP	0.00	[-0.15, 0.14]	0.997
	ET vs. OLP	0.03	[-0.12, 0.18]	0.883
Between-group (T1)	SMT vs. ET	-0.01	[-0.16, 0.14]	0.982
	SMT vs. OLP	0.08	[-0.07, 0.23]	0.434
	ET vs. OLP	0.09	[-0.06, 0.24]	0.335
Within-group (T0–T1)	SMT	-0.10	[-0.24, 0.04]	0.229
	ET	-0.07	[-0.22, 0.07]	0.423
	OLP	-0.02	[-0.16, 0.13]	0.967
Add-on (OLP effect)	(T0–T2) vs. (T0–T1)	0.04	[-0.05, 0.13]	0.413
**Heavy lift SPARC**				
Between-group (T0)	SMT vs. ET	-0.04	[-0.20, 0.12]	0.830
	SMT vs. OLP	0.05	[-0.11, 0.21]	0.769
	ET vs. OLP	0.09	[-0.08, 0.25]	0.418
Between-group (T1)	SMT vs. ET	0.01	[-0.15, 0.16]	0.996
	SMT vs. OLP	0.07	[-0.09, 0.23]	0.564
	ET vs. OLP	0.06	[-0.10, 0.23]	0.615
Within-group (T0–T1)	SMT	-0.10	[-0.23, 0.04]	0.221
	ET	-0.05	[-0.19, 0.09]	0.647
	OLP	-0.07	[-0.21, 0.07]	0.434
Add-on (OLP effect)	(T0–T2) vs. (T0–T1)	-0.01	[-0.10, 0.08]	0.851
**Light lift movement speed (m · s⁻¹)**				
Between-group (T0)	SMT vs. ET	-0.01	[-0.12, 0.10]	0.944
	SMT vs. OLP	0.01	[-0.10, 0.12]	0.978
	ET vs. OLP	0.02	[-0.09, 0.14]	0.862
Between-group (T1)	SMT vs. ET	-0.04	[-0.15, 0.07]	0.706
	SMT vs. OLP	0.05	[-0.06, 0.17]	0.493
	ET vs. OLP	0.09	[-0.02, 0.20]	0.139
Within-group (T0–T1)	SMT	-0.03	[-0.09, 0.03]	0.485
	ET	-0.05	[-0.11, 0.01]	0.122
	OLP	0.01	[-0.05, 0.08]	0.866
Add-on (OLP effect)	(T0–T2) vs. (T0–T1)	0.03	[-0.02, 0.08]	0.254
**Heavy lift movement speed (m · s⁻¹)**				
Between-group (T0)	SMT vs. ET	-0.02	[-0.11, 0.08]	0.904
	SMT vs. OLP	0.04	[-0.05, 0.14]	0.533
	ET vs. OLP	0.06	[-0.04, 0.16]	0.299
Between-group (T1)	SMT vs. ET	0.02	[-0.08, 0.12]	0.867
	SMT vs. OLP	0.03	[-0.07, 0.13]	0.715
	ET vs. OLP	0.01	[-0.09, 0.11]	0.958
Within-group (T0–T1)	SMT	-0.04	[-0.09, 0.01]	0.200
	ET	0.00	[-0.05, 0.05]	1.000
	OLP	-0.05	[-0.10, 0.00]	0.076
Add-on (OLP effect)	(T0–T2) vs. (T0–T1)	-0.02	[-0.05, 0.02]	0.329

Mean differences (Δ) are presented with 95% confidence intervals (CI). Between- and within-group comparisons were estimated using linear mixed models with Tukey adjustment. Within-group changes represent T0–T1 differences. The add-on analysis compared change in SMT/ET + OLP (T0–T2) with SMT/ET (T0–T1) using a linear model with cluster-robust standard errors clustered on participant ID. Bold p-values indicate *p* < 0.05

**Fig. 6:**
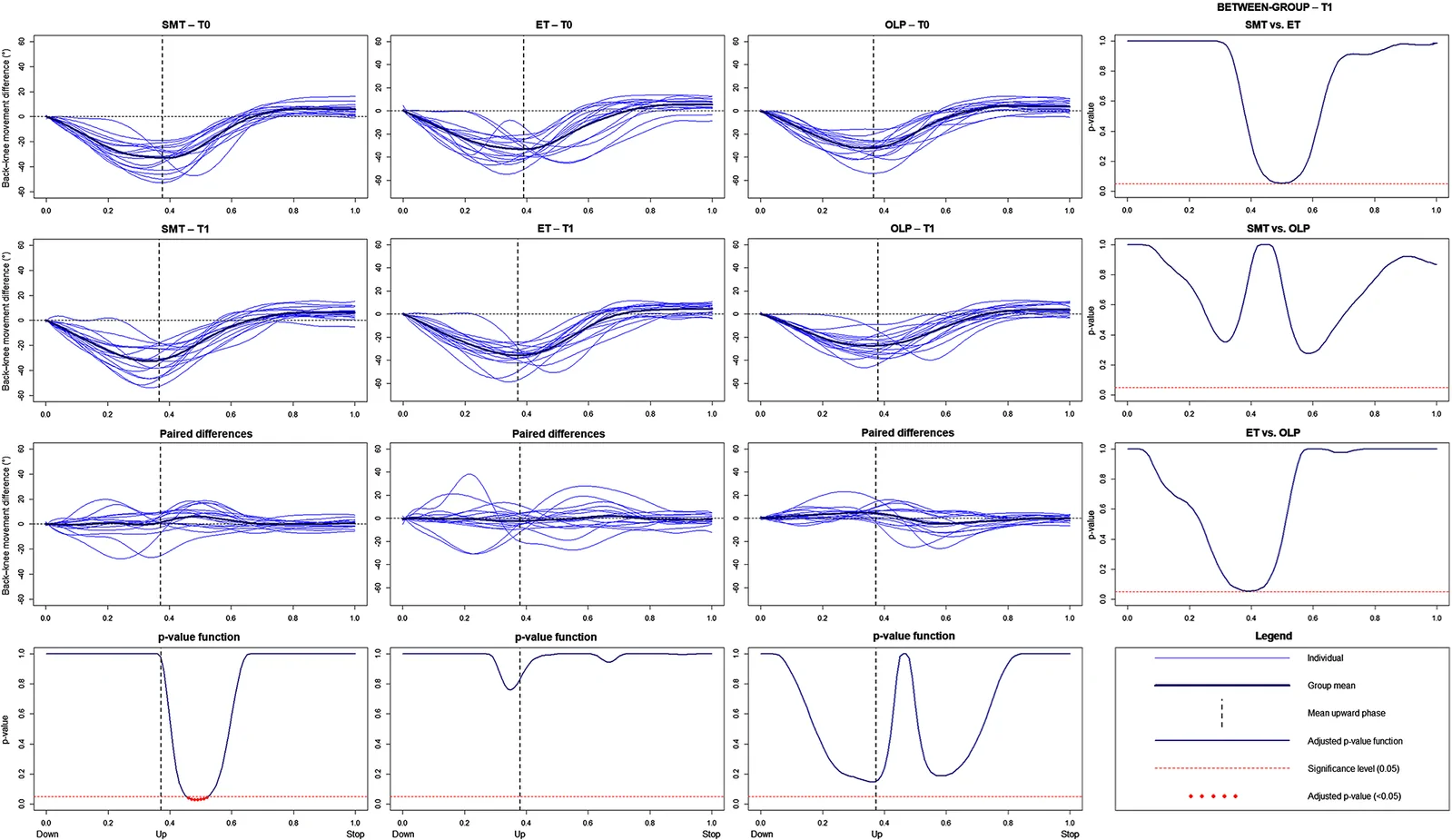
Back-to-knee movement during the light lifting task (FDA). Back–knee movement difference across normalized time (0–1). Within- and between-group comparisons. Positive values indicate greater back movement relative to the knees (back dominance), whereas negative values indicate greater knee movement relative to the back (knee dominance)

FDA of the light lifting task demonstrated a within-group change in intersegmental coordination following SMT, characterized by greater back dominance early in the lifting phase immediately after lift initiation (Fig. 6). This pattern indicates a relatively greater contribution of back movement compared with knee movement during the early part of the lift. No corresponding within-group changes were observed following ET or OLP, and no significant between-group differences were identified.

**Fig. 7:**
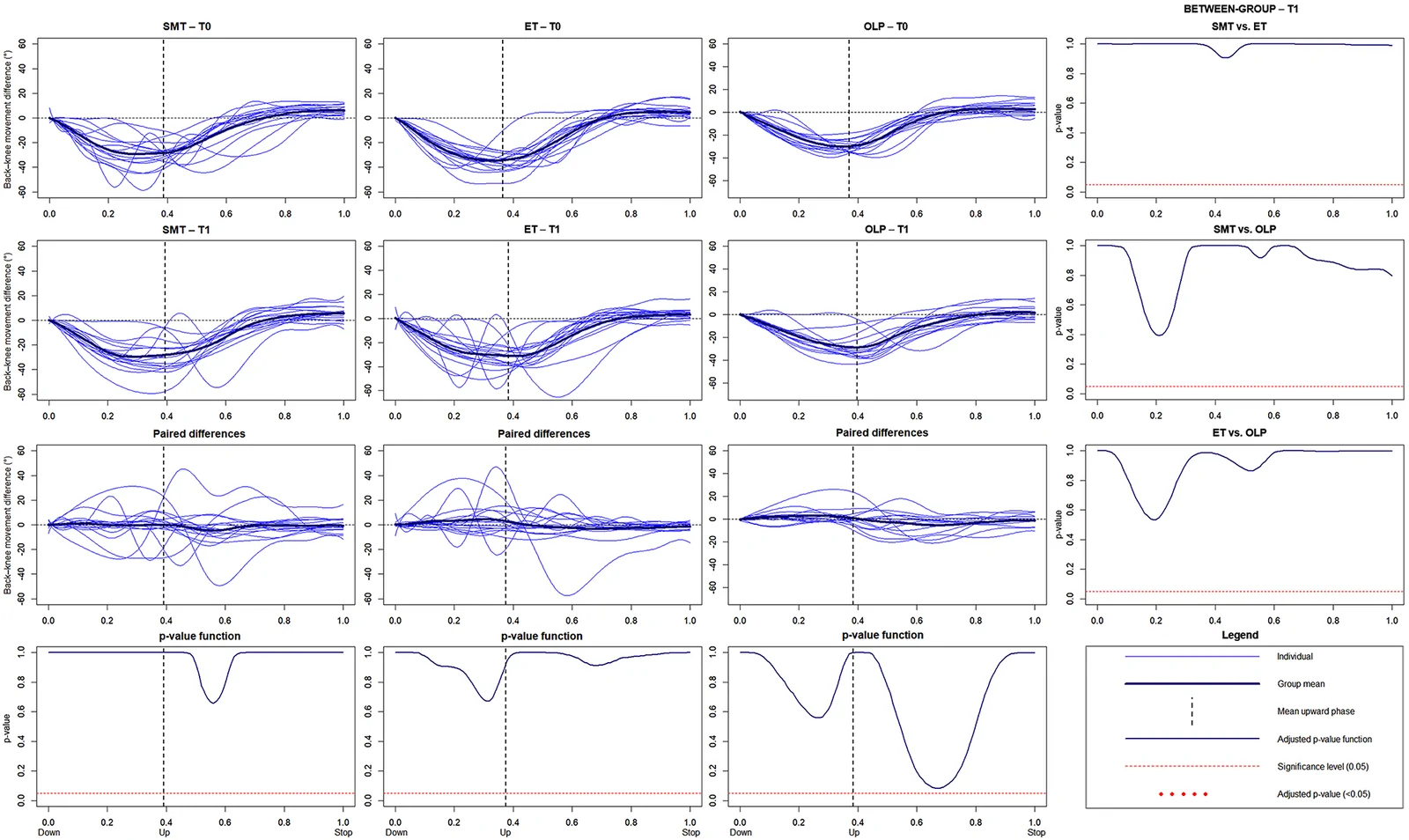
Back-to-knee movement during the heavy lifting task (FDA). Back–knee movement difference across normalized time (0–1). Within- and between-group comparisons. Positive values indicate greater back movement relative to the knees (back dominance), whereas negative values indicate greater knee movement relative to the back (knee dominance)

FDA of the heavy lifting task showed no statistically significant within-group changes in intersegmental coordination following SMT, ET, or OLP (Fig. 7). Likewise, no significant between-group differences were identified, indicating that movement coordination during the heavy lifting task remained stable across interventions.

## Discussion

The primary aim of this study was to investigate the immediate effects of SMT, ET, and OLP on lifting performance and pain in individuals with CBP. Since lifting is a common and functionally relevant everyday task, understanding how these interventions influence movement performance during lifting can be clinically relevant in individuals with CBP. Overall the findings showed no significant changes between the interventions explored in this study. However, the findings did show some within-group changes in lifting performance and pain following the interventions. Within-group changes in movement were also observed in thorax–pelvis tilt and intersegmental coordination, while most conventional discrete biomechanical outcomes showed limited change.

Most spatiotemporal and ROM variables showed limited changes following the interventions, particularly during the heavy lifting condition. Movement smoothness (SPARC) and movement speed remained unchanged across all interventions, reinforcing the overall impression that immediate effects on overall movement performance were minimal. Movement smoothness has been proposed to represent a distinct aspect of movement quality [35], and the absence of changes may indicate relatively stable movement quality already at baseline. More generally, the relatively low baseline pain intensity and disability levels may have limited the potential for improvement following the interventions. However, sensitivity analyses excluding participants reporting no pain at baseline yielded similar results for pain outcomes. The limited immediate changes observed in the present study suggest that lifting patterns associated with CBP may be relatively persistent and not readily altered by short-term interventions, but may instead require several treatment sessions over time.

FDA analyses identified a within-group change in intersegmental coordination following SMT, characterized by a greater relative contribution of the back early in the lifting phase during the light lift. These findings may reflect changes in the coordination and temporal organization of movement following SMT. Previous studies have reported that SMT may affect biomechanical properties such as spinal ROM and spinal stiffness, although findings have been inconsistent [16]. The present results suggest that SMT-related changes during lifting tasks may be more evident in movement coordination than in ROM. Accordingly, analyses that evaluate the full movement trajectory, such as FDA, may help detect subtle changes not captured by conventional discrete biomechanical measures [41]. Together, these findings may indicate that short-term interventions can influence how movements are performed despite limited effects on overall task performance.

In the spatial domain, within-group reductions in thorax–pelvis tilt were observed following OLP during the light lift and following SMT during the heavy lift. Given that thorax–pelvis tilt was used as a proxy for maximum lumbar flexion, these findings may indicate smaller peak sagittal lumbar movement after these interventions. Interestingly, following SMT, this reduction in peak lumbar flexion occurred alongside the altered intersegmental coordination observed in the FDA. Taken together, these findings suggest that SMT did not increase lumbar range of motion, but rather altered the temporal organization and control of the lift. Participants initiated the movement using a more back-dominant strategy early on, while maintaining the lumbar spine in a less flexed position throughout the lift. However, neither finding was accompanied by a significant between-group difference at the primary endpoint, and the add-on analysis did not demonstrate a significant effect of OLP on thorax–pelvis tilt.

The observed differences between the light and heavy lifting conditions may reflect task-dependent constraints on movement adaptability, as heavier loads may be more resistant to change. One possible explanation is that lower-load tasks provide greater opportunity for intervention-related changes in movement performance to emerge, whereas heavier loads constrain lifting strategies due to increased biomechanical demands. Given that human movement is characterized by both consistency and flexibility, enabling adaptation to varying task demands [42], the subtle changes observed may therefore represent short-term adaptations within established movement patterns rather than large-scale biomechanical reorganizations.

These findings may also be interpreted in relation to previous observations that individuals with CBP tend to use less lumbar flexion and adopt a more upright and knee-dominant lifting strategy during lifting tasks [6, 7]. From this perspective, changes in thorax–pelvis movement following SMT and OLP, together with the altered intersegmental coordination following SMT, may be consistent with differences in movement strategies, although whether these changes represent less guarded or more typical movement patterns cannot be determined from the present study. A better understanding of how individuals with CBP perform functional tasks could help distinguish between movement strategies associated with pain-related adaptation and those reflecting more typical movement behaviour. Such insights could contribute to a more objective evaluation of intervention effects and support the development of evidence-based management approaches for CBP.

A within-group reduction in pain was observed following SMT, whereas no clear changes were observed for ET or OLP, and no between-group differences were found. SMT was also ranked highest in perceived effectiveness and willingness to undergo the intervention, followed by ET and OLP. Internal consistency across these measures was low, indicating that they capture related but distinct aspects of the treatment experience. Together, these findings suggest that subjective improvements in pain and treatment evaluation may occur independently of measurable changes in movement performance. This dissociation may suggest that pain reduction and movement performance are at least partly independent constructs in CBP. Movement adaptations in CBP are likely influenced by several interacting factors beyond nociception or pain alone, including learned motor strategies, fear of movement, expectations, and contextual factors. Movement patterns that have developed over time in individuals with CBP may require prolonged or repeated exposure to interventions before measurable changes in movement performance can occur. In addition, considerable individual variability likely exists in how individuals with CBP adapt their movement strategies and respond to different interventions [43].

An important and novel aspect of the present study was the inclusion of OLP. In this study, within-group reductions in thorax–pelvis tilt and increases in hip and knee flexion were observed during the light lift, with significant add-on effects observed for hip flexion in both lifting conditions. However, effects on objective outcomes were inconsistent and not supported by primary between-group comparisons, which aligns with previous evidence suggesting that OLP primarily influences PROMs [25]. Objective biomechanical measures may be less susceptible to expectancy-related influences than PROMs, potentially contributing to the limited observable effects on movement performance. Nevertheless, the findings raise the possibility that expectancy-related mechanisms may contribute to changes in certain aspects of movement performance during lifting tasks in individuals with CBP.

Perceived effectiveness and willingness to undergo the interventions did not align with objective movement changes, emphasizing the importance of integrating subjective and objective outcomes when evaluating treatment responses.

From a clinical perspective, the present findings support the potential value of studying functional movement tasks rather than relying solely on clinical tests or PROMs. Functional movement tasks such as lifting can provide complementary information regarding physical performance during everyday activities. Since lifting is a common and physically demanding daily activity, even subtle changes in movement coordination or quality could potentially have clinical relevance if sustained over time. Compensatory movements may also alter loading patterns and forces acting on adjacent joints and structures. Objective movement analysis, such as motion capture, can therefore contribute to a more comprehensive understanding of physical functioning in individuals with CBP. As more accessible movement-analysis technologies continue to develop, including markerless motion-capture systems, objective movement assessment may become increasingly feasible in future clinical research and practice.

Limitations should be acknowledged. While high-precision motion capture provides detailed biomechanical information, movement performance in a laboratory environment may differ from movement performance in real-world settings. At the same time, the controlled laboratory environment can also be considered a methodological strength, as it enables standardized testing conditions and precise detection of subtle biomechanical changes that may otherwise be difficult to identify in more variable real-world contexts. However, the standardized lifting setup may also have limited natural variation in lifting strategies, potentially reducing the extent of movement changes that could be observed following the interventions. Methodologically, the use of FDA is a strength, as it enables analysis of the entire movement trajectory and detection of time-specific changes that are not captured by discrete measures. Nevertheless, FDA is sensitive to artefacts and marker occlusion, which resulted in exclusion of some trajectories. Finally, the power calculation was based on biomechanical variables from a previous movement laboratory study, whereas additional biomechanical measures such as SPARC and analytical approaches such as FDA were included in the present study. This represents a limitation, as it may have reduced the ability to detect smaller effects in some analyses. In addition, although multiplicity adjustments were applied within relevant comparison families, the number of outcomes and analytical approaches may have increased the overall risk of chance findings. However, only a small number of statistically significant findings were observed across the analyses, with the large majority of comparisons being non-significant.

The study evaluated only immediate effects, and future studies are needed to determine whether the observed biomechanical changes translate into sustained functional improvements over time. However, patients with CBP often seek care with expectations and hope of immediate symptom relief or functional improvement, making the investigation of immediate treatment responses clinically relevant. Immediate responses can nevertheless provide insight into early biomechanical or behavioural adaptations that could become more pronounced following repeated treatment sessions.

## Conclusion

SMT, ET, and OLP demonstrated limited immediate effects on overall movement performance during lifting in individuals with CBP. However, some within-group changes in intersegmental coordination and segment-specific movement patterns were observed, suggesting that certain aspects of movement quality may respond to short-term interventions even when overall movement performance remains unchanged. A within-group reduction in pain was observed following SMT, whereas no clear changes were observed for ET or OLP. Perceived treatment responses did not consistently align with objective movement changes, suggesting the value of integrating both subjective and objective outcomes when evaluating treatment responses. Overall, the clinical relevance of these findings remains uncertain and depends on whether such changes are sustained over time. Future research should therefore focus on longer-term effects to determine whether these immediate responses translate into meaningful clinical improvements.

## Supplementary Information

Below is the link to the electronic supplementary material.

Additional file 1. Participant information

Additional file 2. Randomization

Additional file 3. Patient-reported outcome measures

Additional file 4. SMT dose and treated spinal segments

Additional file 5. ET dose and exercises performed

Additional file 6. OLP information video

Additional file 7. CONSORT 2025 checklist

## Data Availability

De-identified data generated and analyzed during the current study, as well as the statistical analysis code, are available from the corresponding author upon reasonable request. Personal data are not publicly available due to privacy and ethical considerations.

## Abbreviations

CBP: Chronic Back Pain
CONSORT: Consolidated Standards of Reporting Trials
ET: Exercise Therapy
FDA: Functional Data Analysis
HVLA: High-Velocity, Low-Amplitude
LBP: Low Back Pain
NRS: Numerical Rating Scale
ODI: Oswestry Disability Index
OLP: Open-Label Placebo
PROMs: Patient-Reported Outcome Measures
QTM: Qualisys Track Manager
ROM: Range of Motion
SMT: Spinal Manipulation
SPARC: Spectral Arc Length
WHO: World Health Organization
