# Supplementary material for: Immediate effects of spinal manipulation, exercise therapy, and open-label placebo on lifting performance and pain in individuals with chronic non-specific back pain: a motion laboratory-based crossover randomized controlled trial
Source: Chiropr Man Therap. 2026 Oct 14;34:60. doi: 10.1186/s12998-026-00680-w (PMC13670478; doi:10.1186/s12998-026-00680-w)
Supplement: Supplementary file 1 [file 12998_2026_680_MOESM1_ESM.docx]

**PATIENTINFORMATION TILL STUDIEN:**

# ”Omedelbara effekter av spinal ledmanipulation, träningsterapi och öppen placebo hos personer med långvarig ryggsmärta - en laboratoriebaserad randomiserad kontrollerad studie”

Ansvariga forskare:

Wim Grooten, docent, universitetslektor, fysioterapeut och huvudhandledare. Karolinska Institutet

Fredrik Borg, Doktorand och kiropraktor. Lärare vid Kiropraktorhögskolan

Erika Franzén ,Professor, fysioterapeut och bihandledare. Karolinska Institutet

Filip Gedin. PhD, hälsoekonom, Karolinska Institutet.

Förfrågan om deltagande

Vi vill fråga dig om du vill delta i denna studie eftersom du har sökt vård för dina ospecifika långvariga ryggbesvär och din vårdgivare har kontaktat oss, eller så har du själv tagit kontakt med oss.

Titel:

Omedelbara effekter av spinal ledmanipulation, träningsterapi och öppen placebo hos personer med långvarig ryggsmärta - en laboratoriebaserad randomiserad kontrollerad studie

Ansvariga forskare:

Doktorand Fredrik Borg, Handledare: Docent Wim Grooten, Professor Erika Franzén och Med. Dr. Filip Gedin. Karolinska Institutet.

Huvudman

Karolinska Institutet är forskningshuvudman i denna studie. Adressen till Campus Flemingsberg är: Alfred Nobels Allé 23, 141 83 Huddinge.

Bakgrund

Många i Sverige lider av ryggsmärta. Personer med långvariga ryggproblem har ofta svårt att utföra vardagliga rörelser och riskerar att bli frånvarande från arbetet, vilket innebär stora kostnader för individen, arbetsgivaren och samhället. Behandlingen är oftast inriktat på patientens smärta och funktion och kan bland annat bestå utav kiropraktisk behandling och/eller specifika ryggövningar. Studier visar på positiva effekter av båda dessa behandlingar, men de omedelbara effekterna på rörelseutförandet av vardagsrörelser är okända. Dessutom har det varit problematiskt att särskilja effekterna från placebo och fler studier på området ”öppen placebo” har efterfrågats.

I studien studeras tre olika behandlingsmetoder:

- Spinal ledmanipulation (SMT) är en allmän behandlingsmetod för rygg-, nacke- och bäckenproblem och ingår i riktlinjer för behandling av långvarig ryggsmärta. Manipulationsbehandlingen riktas mot ett eller flera segment i ryggraden och/eller bäckenet i den rörelseriktning som terapeuten bedömer vara begränsad. Ledmanipulationen syftar till att normalisera ledrörelse, förbättra funktion och minska smärta.
- Träningsterapi (TT). Dessa övningar är graderade rörlighets-, styrke- och motorkontroll-övningar som utförs i ett smärtfritt rörelseomfång.
- Öppen placebo (ÖP) innebär att man behandlar någon med placebo och samtidigt är helt öppen med detta. Det står ”placebo” på medicinburken. Det kan låta märkligt, men öppen placebo har i tidigare studier visat sig vara effektivt för en rad olika hälso- och smärtproblem och det finns flera positiva exempel på placeboeffekter. Mekanismer bakom placeboeffekten nämns också, men beskrivs dock med en uppriktig ovisshet kring det möjliga utfallet: ”Vi vet inte om behandling med öppen placebo kommer att ha effekt eller inte men vill ge det en chans i den här studien”.

Syfte

Projektet syftar till att studera de omedelbara effekterna av SMT, TT och ÖP på lyftförmåga, gång, framåtböjning och smärta hos personer med långvarig ospecifik ryggsmärta.

Hur går studien till?

Besöket i rörelselabbet inleds med en kort introduktion om upplägget för mätningarna, sedan ber vi dig fylla i ett frågeformulär om dina besvär. Därefter får du byta om till en smidig klädsel, antingen en mjuk helkroppsdräkt eller, om du inte kan eller vill ha dräkten, linne eller t-shirt och shorts/cykelbyxor. När du är ombytt appliceras ett 40-tal mindre reflexmarkörer, som används för att registrera ditt rörelsemönster, på utvalda ställen på huvud, rygg, bål, axlar, armar, händer, höfter, ben, fotleder och fötter. Mätning av rörelsemönster sker såväl före som efter respektive behandling. Du får tre olika typer av behandling i slumpmässig ordning och genomför några vardagsrörelser och rörelsetester. Besöket i rörelselabbet uppgår till cirka 2 timmar. Du får erbjudandet att få följande behandlingar:

- Spinal ledmanipulation: En kiropraktor kommer att utföra en undersökning av hela ryggen för att identifiera de leder med begränsad rörelse. Vanlig kiropraktisk ledmanipulation av dessa områden tillämpas som syftar till att förbättra funktionen och minska smärtan. Undersökning och behandling tar cirka 5 minuter totalt.
- Träningsterapi: Efter en kort genomgång ber vi dig att utföra tre standardövningar som syftar till att öka rörligheten i ryggen och den motoriska kontrollen och koordinationen av musklerna som kontrollerar ländryggen, bäckenet och höftlederna i tre rörelseriktningar. Du väljer själv den svårighetgrad, intensitet och antal repetitioner som passar dig. Övningarna tar totalt cirka 5 minuter att utföra.
- Öppen placebo: Du kommer att se ett kort nyhetsreportage (1 minut 25 sekunder), där en deltagare i en ÖP-studie intervjuas om de upplevda positiva effekterna av ÖP behandlingen. Forskarna förklarar att i) en placeboeffekt kan vara kraftfull, ii) att kroppen automatiskt kan svara på att ta placebo-piller som Pavlovs hundar som saliverade när de hörde en klocka, iii) att en positiv attityd kan vara till hjälp, men är inte nödvändigt, iiii) att placebopiller kan ha en effekt även om du vet om att det är placebo och inte tror på det. Efter det erbjuds du två placebopiller som enkelt går att svälja med vatten. Pillren innehåller den inaktiva substansen mikrokristallin cellulosa från en burk tydligt märkt med "placebo". Du behöver inte ta dessa piller om du inte vill. Hela behandlingen tar cirka 5 minuter.

Vi kommer att fråga dig om att utföra några enkla rörelser/vardagsaktiviteter så att vi kan registrera hur ditt individuella rörelsemönster ser ut och om det ändrats efter att du har fått respektive behandling:

- Lyft av låda. Du kommer att få lyfta en plastback från golvet till ett bord framför dig. Du ska välja en lätt vikt, samt en tyngre vikt som du bedömer att du skulle kunna lyfta. Vi ber dig att lyfta backen från golvet till bordet i självvald bekväm hastighet ett antal gånger med vila emellan varje lyft. Samtidigt du utför lyften, så registreras dina rörelser.
- Gång. Du kommer att gå cirka 10 meter framåt i självvald bekväm hastighet ett antal gånger och kan vila emellan om det behövs. Samtidigt du går, så registreras dina rörelser.
- Aktiv rörlighet. Vi kommer med en filtpenna rita ett litet sträck på din rygg för att kunna mäta din ländryggsrörlighet. Vi ber dig att böja dig framåt så långt du kan med raka ben och stanna där i några sekunder, så avståndet från dina fingertoppar till golvet kan registreras och vi kommer även att mäta hur många centimeter som sträcket på din ländrygg har förflyttat sig. Vi ber dig utföra denna rörelse ett antal gånger och torkar bort sträcket igen med en tygtrasa efter sista mätningen.

Vilka är riskerna?

Inga kända risker föreligger.

Behandlingarna SMT och TT är vanliga behandlingar och är standardbehandling vid denna typ av besvär. SMT behandling sker under kontrollerade förhållanden och utförs av en legitimerad Kiropraktor. Vid TT får du själva välja antal gånger, hur länge du vill utföra övningen och du väljer även själv intensiteten i övningen, allt för att minska risken för eventuell överbelastning. Övningarna är kroppsegna övningar och inga externa vikter kommer att användas. ÖP piller innehåller overksamma substanser som tidigare inte har gett några (allergiska) biverkningar.

Vid applicering av markörerna används kardborrar om du har den smidiga dräkten på dig, alternativt dubbelhäftad tejp som är allergitestad och används för applicering av tupéer. Detta har tidigare inte lett till några allergiska hudreaktioner i vårt rörelselaboratorium.

Testerna och rörelserna som används för utvärdering är enkla, kontrollerade och säkra. Vid lyft av låda väljer du själv belastningsnivån och det är endast några lyft som skall genomföras. Du får vila mellan lyften för att minska risken för överbelastning. Gång och framåtböjning av ryggen är säkra kliniska tester och sker under kontrollerade former. Du behöver inte få spinal ledmanipulation, utföra övningar eller ta placebopiller om du inte vill och kan avstå från att utföra teströrelserna. Du behöver inte ange några skäl för att inte delta i behandlingarna eller testerna, men vi erbjuder samtalsmöjlighet ifall du vill prata om det.

Finns det några fördelar?

Fördelar med att delta är dels att du får undersökning och behandling för dina symptom. Du får även en objektiv bedömning av hur dina besvär påverkar dina vardagsrörelser, samt en noggrann och avancerad mätning av din aktiva rörlighet – något som sällan erbjuds vid vanlig behandling. De övningar som testats kan med fördel användas som hemövningar och tidigare studier har visat att dessa är säkra och effektiva.

Varför vill vi utföra studien?

Vad som är oklart i forskningen är hur stora de omedelbara effekterna av behandlingarna är, och hur de kan påverka inte bara smärta utan även rörelsemönstret i vardagsrörelser. Vidare är det oklart om det finns eventuella synergieffekter av kombinationsbehandling och huruvida en placebobehandling har effekt.

Hantering av data och sekretess

All data hanteras av forskarna i lösenordskyddade datorer och servrar med full sekretess. Dina svar och dina resultat kommer att behandlas så att inte obehöriga kan ta del av dem. Dina personuppgifter behandlas av projektet i forskningssyfte enligt EU:s dataskyddsförordning (GDPR) utifrån den rättsliga grunden att utföra en uppgift av allmänt intresse.

Vi använder uppgifterna vi samlar in om dig för att kunna analysera ditt specifika rörelsemönster utifrån dina angivna besvär och demografiska uppgifter. Vi kommer att ge varje person en kod så att dina personuppgifter inte längre kan kopplas till dig som person. Detta kallas för ”pseudonymisering”. Dina uppgifter sparas i cirka 15 år (minst 10 år efter publicering). Dessa data kommer att förvaras omsorgsfullt åtskilda från personuppgifter. Dina pseudonymiserade uppgifter kan komma att delas med närstående forskare. Du har rätt att en gång årligen begära ett registerutdrag på de uppgifter som Karolinska Institutet (KI) lagrar om dig. Du har även rätt att begära rättelse av uppgifterna eller få dem raderade. Kontakta i så fall huvudansvarig forskare. Om du har synpunkter på behandlingen av dina personuppgifter kan du vända dig till KI:s dataskyddsombud på [dataskyddsombud@ki.se](mailto:dataskyddsombud@ki.se). Om du inte är nöjd med KI:s svar, kan du vända dig till Integritetsskyddsmyndigheten med klagomål imy@imy.se eller 08-657 61 00

Hur får jag information om studiens resultat?

Deltagarna får ta del av sina individuella resultat, samt resultatet av hela studien efter analysfasen. Rapporten kommer att presenteras i vetenskaplig tidskrift, konferens och forskningsseminarier samt förhoppningsvis i en doktorsavhandling. Kontakta ansvariga forskare om du vill ha gruppens eller dina resultat (se nedan).

Försäkring och ersättning

Deltagarna omfattas av Karolinska Institutets samlingsförsäkring inom SPS (särskilt personskadeskydd) vid Kammarkollegiet. Det utgår ingen ersättning för deltagande i projektet.

Frivillighet

Ditt deltagande i projektet är frivilligt och du kan när som helst, utan särskild förklaring, välja att avbryta. Detta kommer inte att påverka din sedvanliga behandling eller omhändertagande. Eventuella data som har samlats in kommer då att raderas.

Samtyckesformulär

Du har genom detta informationsblad informerats om studien, och har även fått möjlighet att ställa muntliga frågor. Genom att underteckna samtyckesblanketten ges deltagande i studien.

Ansvariga

Karolinska Institutet är forskningshuvudman för denna studie och därmed personuppgiftsansvarig för behandlingen av dina personuppgifter. Din kontaktperson är: Wim Grooten, docent. [Wim.Grooten@ki.se](mailto:Wim.Grooten@ki.se); telefon 08-52488861. Forskningen utförs av doktoranden Fredrik Borg, [fredrik.borg@ki.se](mailto:fredrik.borg@ki.se).

Omedelbara effekter av spinal ledmanipulation, träningsterapi och öppen placebo hos personer med långvarig ryggsmärta - en laboratoriebaserad randomiserad kontrollerad studie.

**SAMTYCKE TILL ATT DELTA I FORSKNINGSSTUDIEN**

Jag har fått ta del av den skriftliga/muntliga information om denna forskningsstudie och har haft möjlighet att ställa frågor. Jag får behålla den skriftliga informationen.

Jag samtycker att delta i forskningsstudien,

Plats: ___________________________________________________ Datum: ______________

Underskrift: _______________________________________________________

Namnförtydligande: _________________________________________________
