# Supplementary material for: Immediate effects of spinal manipulation, exercise therapy, and open-label placebo on lifting performance and pain in individuals with chronic non-specific back pain: a motion laboratory-based crossover randomized controlled trial
Source: Chiropr Man Therap. 2026 Oct 14;34:60. doi: 10.1186/s12998-026-00680-w (PMC13670478; doi:10.1186/s12998-026-00680-w)
Supplement: Supplementary file 2 [file 12998_2026_680_MOESM3_ESM.docx]

SUBJECT# _______________

**PAIN**
BASELINE

POST INTERVENTION 1

POST INTERVENTION 2

POST INTERVENTION 3

**EFFECTIVENESS AND WILLINGNESS**
SMT

ET

OLP

 **RANK THE INTERVENTIONS** (USE EACH INTERVENTION ONLY ONCE)
 **1: ________________________

2: ________________________

3: ________________________**
